# The Prevalence and Impact of Bacteremia Among Neonates Receiving Parenteral Nutrition: A Multicenter Retrospective Study from Saudi Arabia

**DOI:** 10.3390/pharmacy14010017

**Published:** 2026-01-26

**Authors:** Shaker Althobaiti, Aisha H. Alshehri, Abeer K. Alorabi, Alhussain Alzahrani, Lama Marwan Fetyani, Ebtihal Mohsin Fairaq, Enas Ahmed Abukwaik, Njood Abdulsalam Alharbi, Abrar A. Alotaibi, Safia Ghali Alotibi, Shaimaa Alsulami, Abdullah Althomali, Ahmed Ibrahim Fathelrahman

**Affiliations:** 1 AlHada Armed Forces Hospital, Taif 26792, Saudi Arabia; ab.99@windowslive.com (A.A.A.); alotibi.s.pharmd@gmail.com (S.G.A.); 2 King Fahad Armed Forces Hospital, Jeddah 23311, Saudi Arabia; dr.aisha.alshehri@gmail.com (A.H.A.); alh.1988@hotmail.com (A.A.); lamafetyani@gmail.com (L.M.F.); ebtihal.fairaq@gmail.com (E.M.F.); drenaskwaik@gmail.com (E.A.A.); alharbi.njood@hotmail.com (N.A.A.); 3 King Faisal Medical Complex (KFMC), Taif 26514, Saudi Arabia; dr.abeer1993@outlook.sa (A.K.A.); phd-a2009@hotmail.com (A.A.); 4 R2 Clinical Pharmacy, King Abdulaziz University Hospital, Jeddah 22252, Saudi Arabia; 5 Department of Pharmacy Practice, College of Pharmacy, Umm Al-Qura University, Makkah 24381, Saudi Arabia; saalsulami@uqu.edu.sa; 6 Department of Clinical Pharmacy, College of Pharmacy, Taif University, Taif 21974, Saudi Arabia

**Keywords:** parenteral nutrition, neonates, bacteremia, Saudi Arabia, multidrug resistance bacteremia, mortality rate

## Abstract

(1) Background: We aimed to determine rates of bacteremia and multidrug resistance (MDR) bacteremia and associated risk factors among neonates receiving parenteral nutrition (PN). (2) Methods: This is a multicenter study conducted in three neonatal intensive care units in Saudi Arabia, including 414 neonates who received PN. Associations were assessed using Chi-square or Fisher’s Exact tests when applicable and logistic regression analyses were conducted to determine factors predicting outcomes. Odds ratios with their 95% confidence intervals were computed, and a *p* value < 0.05 was considered statistically significant. (3) Results: PN was started within the first 10 days of life in 74.4% of cases. Fat emulsion was administered to 38.9% of the newborns. Blood cultures were positive in 24.9% of patients. Among the positive cultures, 4.9% were confirmed to have MDR bacteria. The mortality rate following bacteremia was 7.8%. The use of fat emulsion (*p* = 0.003), birth weight < 700 g (*p* < 0.001), and a gestational age within 27 weeks (*p* < 0.001) predicted bacteremia. (4) Conclusions: There was an association between the PN and bacteremia. Significant predictors of bacteremia were the use of fat emulsion, birth weight < 700 g, and a gestational age within 27 weeks.

## 1. Introduction

Parenteral nutrition (PN) is a critical intervention for neonates who are unable to receive adequate enteral nutrition. While PN supports essential growth and development, it also carries risks including infections and the potential development of multidrug-resistant (MDR) bacterial infections [1,2,3]. For example, Turel et al. reported an outbreak of *Achromobacter xylosoxidans* at a neonatal intensive care unit in Turkey [4]. Endotracheal intubation, intravenous catheter use, total parenteral nutrition, and prolonged antibiotic therapy were the predisposing conditions. Aldemir et al. compared the characteristics of neonates diagnosed with sepsis at a sentinel neonatal unit in Istanbul, Turkey, to those without sepsis in order to determine risk factors [5]. Presence of central venous catheter, ventilation support, total parenteral nutrition, and prolonged hospitalization were risk factors for sepsis development. Perlman et al. compared the risk factors for catheter-related versus non-catheter-related bloodstream infections (BSIs) among infants hospitalized in two neonatal intensive care units in New York City [6]. In neonates with a central catheter, PN was a significant risk factor for BSI (RR: 4.69, 95% CI: 2.22, 9.87). Al-Matary et al. reported a significant association between total parenteral nutrition use, surgical insertion of a central line, inotrope use, and spontaneous intestinal perforation and coagulase-negative staphylococci infection among neonates admitted to a neonatal intensive care unit in Riyadh, Saudi Arabia [7].

According to Hoseini et al., there was a statistically significant relationship between invasive procedures (such as umbilical catheters, central venous catheters, surgery, and TPN) and sepsis (*p* = 0.001) among neonates admitted to three neonatal intensive care units in northwest Iran [8]. Valdés-Corona et al. from Mexico reported a case series of extraordinarily isolated Gram-negative bacteria associated with parenteral nutrition use [9].

Understanding the clinical outcomes and risk factors associated with PN is crucial for improving neonatal care. This study aimed to determine the rates of bacteremia and multidrug resistance bacteremia among neonates receiving PN, assess their clinical outcomes, and determine associated risk factors.

## 2. Materials and Methods

**Study design and setting:**

This was a multicenter cross-sectional study conducted from January 2023 to December 2023 in three tertiary care hospitals in Saudi Arabia, involving 414 neonates who received PN in a neonatal intensive care unit (NICU). Data was obtained retrospectively from the medical records of the three hospitals.

**Inclusion criteria:**

The inclusion criteria include all NICU patients who received PN for 3 days or more.

**Primary and secondary measured patients’ outcomes:**

The primary outcome was to assess the prevalence of bacteremia (positive blood culture) among neonates who require nutritional support. Blood cultures were obtained when signs of sepsis appeared, as directed by the physician’s judgment. The secondary outcomes were 30-day mortality, the occurrence of multidrug resistance, and exploring risk factors that would contribute to bacteremia in PN-receiving neonates.

**Ethical approval:**

The study was approved by the Scientific Research Ethics Committee in King Faisal Medical Complex (KFMC) in Taif (reference number: 2023-B-9) and the Research Ethics Committee of Armed Forces Hospitals-Jeddah (reference number: REC 559).

**Data categorization:**

Data was first obtained in an Excel sheet and then transferred into the IBM Statistical Package for Social Sciences (SPSS) version 26.0 for analyses. Gestational age was categorized according to the three hospitals’ policy into three groups: <26 weeks, 26–27 weeks, and >27 weeks. Later on, the first two groups were combined together and considered as within 27 weeks to reflect the observed significant pattern. Similarly, birth weight was categorized into three groups: <700 g, 700–1200 g, and >1200 g. The age when PN was started was divided into three groups: ≤10 days, 11–28 days, and >28 days.

**Statistical analysis:**

Variables were analyzed first descriptively, and the findings were presented as frequencies and percentages. Associations were assessed using Chi-square and Fisher’s Exact tests where appropriate, and multiple logistic regression analyses were used to predict the factors associated with the outcomes. A *p* value of <0.05 was considered statistically significant and the odds ratios (ORs) were computed together with their 95% confidence intervals.

## 3. Results

Table 1 shows the general characteristics of 414 neonates. The majority of newborns (65.5%) were born after 27 weeks of gestation, and most of them had a birth weight over 1200 g (58.7%). PN was started within the first 10 days of life in 74.4% of cases. Female patients accounted for 53.9% of the included cases, while males comprised 46.1%. Peripheral vascular devices were predominantly used by 44.7%, followed by central vascular devices (39.1% of cases). Fat emulsion was administered to 38.9% of the newborns.

Blood cultures showed no growth in 75.1% of cases, while 24.9% were positive. Among the positive cultures, 4.9% were confirmed to have MDR bacteria (i.e., 1.2% of all neonates). The mortality rate among all neonates was 4.3% (i.e., 18 cases out of 414 neonates) and 7.8% following bacteremia (i.e., 8 cases out of 103 neonates). Among the 18 neonatal deaths who received PN, 4 (22.2%) were directly linked to bloodstream infections such as bacteremia, sepsis, line infection, or CLABSI. The other 14 deaths (77.8%) resulted from different causes, which were not specified as they fell outside the scope of this study.

The female gender was significantly (*p* value < 0.001) more frequently present in the positive bloodstream culture group (69.9%) compared to the no-growth group (48.6%), in contrast to the male gender, which represented 51.4% of the no-growth group and 30.1% of the positive blood culture group (Table 2). A gestational age of <26 weeks (*p* value < 0.001), birth weight of <700 g (*p* value < 0.001), use of a central vascular device (*p* value < 0.001), use of fat emulsion (*p* value < 0.001), and the presence of multidrug resistance (*p* value = 0.015) were all significantly associated with a higher probability of having positive blood cultures than their counterparts. The cause of death among the positive blood culture group was more likely due to bacteremia, line infection, or CLABSI (*p* value = 0.023).

Table 3 shows correlates of mortality. Only use of a central vascular device (*p* value = 0.032), fat emulsion (*p* value = 0.048), and multidrug resistance (*p* value < 0.001) were significantly associated with mortality. Table 4 shows correlates of multidrug resistance. Only birth weights of ≤1200 g (*p* value = 0.008), positive bloodstream culture (*p* value = 0.015), and mortality (*p* value < 0.001) were significantly associated with multidrug resistance.

In multivariate analyses (Table 5), significant independent associations were found between bacteremia and the use of fat emulsion (*p* = 0.003), birth weight < 700 g (*p* < 0.001), and a gestational age within 27 weeks (*p* < 0.001). None of the variables were found to be significant independent predictors of mortality or multidrug resistance, and thus, findings were not presented.

## 4. Discussion

PN use and the administration of a surgically inserted central line are each independent risk factors for infections among neonates, and the presence of both together increases this risk [7]. Evidence from the literature explains that bacteremia occurs among neonates receiving PN by contamination of infusates during the preparation of the solution or infected catheters during catheterization due to poor handling [3]. Skeath and Banait argue that some contaminations in PN solutions occur at the stage when the fat-soluble vitamins are added to intravenous lipids on the day of use to avoid instability issues [2]. Our current study aimed to determine the rates of bacteremia and multidrug-resistant bacteremia among neonates receiving PN, assess their clinical outcomes, and determine associated risk factors.

### 4.1. The Indications and Timing of PN Adminstration

According to the Irish National Clinical Practice Guidelines on the use of PN in neonatal and pediatric units, the intake of sufficient nutrition is highly essential for infants and children, more so than for adults, to maintain body tissues and promote growth, particularly in preterm infants, for whom starvation for even a day is extremely harmful [10]. PN is absolutely indicated in some cases, such as functional immaturity of the GIT (e.g., preterm infants < 32 weeks’ gestation or weighing < 1.5 kg), intestinal failure, post-GIT surgery, necrotizing enterocolitis, and congenital GIT defects, and it is also relatively indicated in other cases [10]. Guidelines established worldwide by ministries of health in high- and upper-middle-income developing countries were quite consistent regarding the indications and the timing for initiating PN for neonates [11,12,13]. Our current study did not capture the indications for the initiation of PN in the studied sample. However, the current practice adopted by the Saudi Ministry of Health is consistent with the international guidelines regarding PN indications including the following: when GIT is not functional or cannot be accessed, or when nutrient needs exceed what can be provided through enteral nutrition (EN); in case of intestinal failure (e.g., pseudo-obstruction, short bowel); post-GIT surgery; necrotizing enterocolitis; congenital GIT defects; and critically ill patients when EN is unable to meet energy requirements for energy expenditure and growth [11]. The American Society for Parenteral and Enteral Nutrition recommends the prompt initiation of PN after birth as soon as appropriate vascular access is obtained [14]. The Saudi guidelines stated different time frames for initiating PN that are specific to different situations, ranging from within 8 h (for very-low-birth-weight infants (<1500 g) or those with a gestational age < 32 weeks) to after 2–3 days (for full-term babies > 37 weeks or weight > 2500 g if they are expected to achieve full enteral feed within 5 days) [11]. The majority of neonates (74.4%) in our current sample involved cases where the ages were ≤10 days when PN was started.

### 4.2. Rates of Bacteremia

Our current study revealed a 24.9% rate of bacterial growth, with 4.9% of them confirmed as MDR bacteria (i.e., 1.2% of all neonates). An old study from the Netherlands [1], dating back to 1988, documented extremely high rates of clinical sepsis and bacteriologically confirmed sepsis among neonates receiving PN (approximately 59% and 16.8%, respectively).

### 4.3. Patients’ Clinical Outcomes

According to Zingg et al., the nosocomial bacteremia-attributable mortality rate among neonates receiving PN is 11% [3]. The attributable mortality rate reflects the rates occurring purely due to bacteremia. This means the observed rates among such a group of patients would be higher, as they include, in addition to the bacteremia-attributable rates, rates due to other factors. In Turkey, the study by Turel and associates revealed a 13.6% mortality rate [4]. Another Turkish study by Aldemir et al. revealed mortality rates of 19.8% and 4.1% among early-onset sepsis and late-onset sepsis neonates, respectively [5]. In our study, the mortality rate following bacteremia was 7.8% (i.e., 4.3% of all neonates), and four of these cases (i.e., 22.2% of deaths) were due to bacteremia, line infection, or CLABSI. Thus, the bacteremia-attributable mortality rate among our patients can be considered 4.6% (i.e., 7.8–3.2%). Such rates are lower than those from the literature.

### 4.4. Factors Associated with Bacteremia

According to Perlman et al., a low birth weight < 1000 g, central venous catheter, and PN use were all significantly more prevalent among neonates with bacteremia than among those without infections [6]. The study by Beganovic et al. revealed an association between sepsis rates and the duration of PN therapy, where those receiving PN for longer periods tend to experience higher rates of sepsis [1]. In our study, the correlates of bacteremia, based on univariate analysis, were female gender, gestational age of <26 weeks, birth weight of <700 g, use of central vascular device, use of fat emulsion, and the presence of multidrug resistance. According to Comerlato et al., longer hospitalization time, longer PN time, and longer catheter time were found to be associated with CLABSI [15]. According to Pitiriga et al., the duration of central venous catheter placement remains an important risk factor for CLABSIs in hospitalized patients [16]. The higher rates of bacteremia observed among neonates treated using a central line might be explained by the association between the use of a central line and the long duration of treatment. Lipids are important for neonatal parenteral nutrition. However, fewer than half of the patients in our study received intravenous fat emulsions. All neonates in our study received parenteral nutrition with carbohydrates and amino acids. The possible reasons for not receiving intravenous lipid emulsions might include presence of clinical contraindications such as suspected or confirmed sepsis, hypertriglyceridemia, hepatic dysfunction, or cholestasis. For this study, patients were classified based on the actual PN components they received. This enabled us to examine whether lipid administration itself might be linked to a higher risk of bacteremia—a connection suggested by previous research. It has been argued that intravenous lipid emulsions can promote microbial growth or increase the risk of infection in neonates. However, this argument can be considered a controversial area as some scholars and expert practitioners raise objections against it [17]. The findings of our current data support the presence of an association between the use of fat emulsion and the occurrence of bacteremia. However, due to the retrospective nature of the data (i.e., possibility of missing or inaccurate information) and the cross-sectional design (i.e., simultaneous measurement of variables and absence of temporal relationship), we cannot assume a causative role of the fat emulsion, and possible confounding variables may have some effects; thus, such findings require further investigations. The incidence of potential infections from these solutions increases with time as well. In this population, the infusion of lipids was administered at rates ranging from 12 to 20 h for all patients receiving the fat emulsion.

Deaths among the positive blood culture group were more likely due to bacteremia, bloodstream infection, line infection, or central line-associated bloodstream infections (CLABSIs). On the other hand, the correlates of mortality were the use of a central vascular device, fat emulsions, and multidrug resistance. The correlates of multidrug resistance were birth weight ≤ 1200 g, positive bloodstream culture, and mortality.

Based on multivariate analyses, significant independent predictors of bacteremia were the use of fat emulsion (*p* = 0.003), birth weight < 700 g (*p* < 0.001), and a gestational age within 27 weeks (*p* < 0.001). Logistic regression models in Perlman et al.’s study proved that the low birth weight and central venous catheter were among the significant predictors of bacteremia [6].

The current study was able to provide a baseline estimate of bacteremia occurring among neonates receiving PN therapy in three tertiary hospitals in Saudi Arabia. In addition, the study provided an overview of patients’ outcomes including mortality and the occurrence of multidrug resistance. Finally, the study was able to determine the factors associated with bacteremia, multidrug resistance, and mortality. A strength of the study is that the sample was from three hospitals. This provides a better representation of the population at least in the western region of Saudi Arabia. The sample size was larger than some published studies and case reports focusing on bacteremia among neonates in general or neonates receiving PN therapy [4,9]. The larger studies in the literature covered all hospitalized neonates and then counted the numbers with bacteremia, septicemia, or sepsis and included PN treatment as a possible factor [5,6,7,8]. Cases from different studies differed in the type of bacteria responsible for the infection and, potentially, in the available treatment facilities; thus, outcomes differed [1,4,5,6,7,9]. However, overall, our findings can be considered consistent with the published literature and supportive of the available evidence on bacteremia associated with PN use among neonates. Our study has limitations: it included all neonates who received PN for more than 3 days for various reasons but did not record the reasons for initiating PN or their baseline clinical conditions. This makes it difficult to assess how disease severity influences infection risk. In addition, laboratory data are usually documented electronically or occasionally handwritten and separated from the patients’ records from which the current data were collected. So, we were unable to identify the isolated bacterial species in the present study.

Knowing the risk of bacteremia among populations receiving PN and their outcomes is crucial for establishing protective measures, adopting required policies and setting the standards of care that protect health and save lives. Establishing the baseline data is useful for monitoring the progress in this regard and for benchmarking. A variety of helpful suggested practices have been published in the literature. According to Opilla, adherence to evidence-based practices for inserting catheters, chlorhexidine skin preparation, and meticulous care of the intravenous site can reduce the risk of catheter-related bloodstream infections. Catheter lock therapy, daily evaluation of continued need for PN, using enteral instead of PN support, glucose control, and avoiding overfeeding can reduce the risk for complications even further [18]. A collaborative task force of eminent European and Chinese pediatric nutrition societies developed evidence-based guidelines for pediatric PN for healthcare professionals [19]. Among their recommendations were recommendations related to the stability of PN solution, drug compatibility, light protection, and vitamin stability. Those include the following examples: “(1) The appropriate measures to secure the catheter in place and education for users on correct maintenance and safety of the catheter. (2) PN should be administered wherever possible using an admixture formulation validated by a licensed manufacturer or suitably qualified institution. (3) Alternative ingredients should not be substituted without expert advice or repeated validation. (4) Mixing of medications with PN in administration lines should be avoided unless validated by the manufacturer or accredited laboratory. (5) Multi-layer bags which are impermeable to oxygen are recommended for PN administration”. The authors of the current paper recommend that hospital nurses should be trained in various practices approved by guidelines when dealing with PN. In addition, PN administration should be documented in the patients’ records, particularly whenever a fever or other symptoms indicate the possible presence of bacteremia. In the same context, pharmacists involved in providing care or services related to PN therapy should be offered adequate training. Katoue reviewed the different roles played by the pharmacists in this important and complex area [20]. Roles included “assessment of patients’ nutritional needs; the design, compounding, dispensing, and quality management of PN formulations; monitoring patients’ response to PN therapy; supervision of home parenteral nutrition (HPN) programs; education of patients, caregivers, and other healthcare professionals on nutrition support and conducting PN-related research and quality improvement activities”.

## 5. Conclusions

Our study showed there was an association between the PN and bacteremia. There were different contributing factors that could lead to this result including fat emulsion, route of administration, infants’ low weight, and gestational age. Hospital-based resistant organisms suggest that line contamination may also contribute to infections. A larger future study using standardized indications and severity assessments, and identifying those needing extra care before PN, is needed.

## Figures and Tables

**Table 1:** General characteristics of the 414 neonates who received parenteral nutrition.

Variable	Categories	Frequency (%)
Sex	Female	223 (53.9)
	Male	191 (46.1)
Gestational age	<26 weeks	101 (24.4)
	26–27 weeks	42 (10.1)
	>27 weeks	271 (65.5)
Birth weight	<700 g	78 (18.8)
	700–1200 g	93 (22.5)
	>1200 g	243 (58.7)
Age when PN was started	≤10 days	308 (74.4)
	11–28 days	62 (15.0)
	>28 days	44 (10.6)
Vascular device	Central	162 (39.1)
	Peripheral	185 (44.7)
	Peripheral changed to central	60 (14.5)
	Central changed to peripheral	7 (1.7)
Fat emulsion	No	253 (61.1)
	Yes	161 (38.9)
Bacteremia	Negative	311 (75.1)
	Positive	103 (24.9)
Multidrug resistance (MDR)	No	409 (98.8)
	Yes	5 (1.2)
Mortality	No	396 (95.7)
	Yes	18 (4.3)
Cause of death *	Other reasons	14 (77.8)
	Bacteremia/sepsis/line infection/CLABSI	4 (22.2)

* i.e., out of 18.

**Table 2:** Correlates of positive bloodstream culture among the 414 admitted neonates.

Variable	Categories	Bloodstream Culture		
		No Growth (n = 311) F (Column%)	Positive (n = 103) F (Column%)	*p* Value
Sex	Female	151 (48.6)	72 (69.9)	<0.001
	Male	160 (51.4)	31 (30.1)	
Gestational age	<26 weeks	14 (4.5)	87 (84.5)	<0.001
	26–27 weeks	33 (10.6)	9 (8.7)	
	>27 weeks	264 (84.9)	7 (6.8)	
Birth weight	<700 g	6 (1.9)	72 (69.9)	<0.001
	700–1200 g	72 (23.2)	21 (20.4)	
	>1200 g	233 (74.9)	10 (9.7)	
Age when PN was started	≤10 days	231 (74.3)	77 (74.8)	0.158
	11–28 days	51 (16.4)	11 (10.7)	
	>28 days	29 (9.3)	15 (14.6)	
Vascular device	Central	82 (26.4)	80 (77.7)	<0.001
	Peripheral	165 (53.1)	20 (19.4)	
	Peripheral changed to central	57 (18.3)	3 (2.9)	
	Central changed to peripheral	7 (2.3)	0	
Fat emulsion	No	231 (74.3)	22 (21.4)	<0.001
	Yes	80 (25.7)	81 (78.6)	
Multidrug resistance	No	310 (99.7)	99 (96.1)	0.015 *
	Yes	1 (0.3)	4 (3.9)	
Mortality	No	301 (96.8)	95 (92.2)	0.089 *
	Yes	10 (3.2)	8 (7.8)	
Cause of death	Other reasons	10 (100)	4 (50.0)	0.023 *
	Bacteremia/sepsis/ line infection/CLABSI	0	4 (50.0)	

* Fisher’s Exact test; other analyses were performed using the Chi-square test, and statistically significant results are indicated by bold font. F: Frequency.

**Table 3:** Correlates of mortality among the 414 admitted neonates.

Variable	Categories	Mortality		
		No (n = 396) F (Column%)	Yes (n = 18) F (Column%)	*p* Value
Sex	Female	213 (53.8)	10 (55.6)	0.883
	Male	183 (46.2)	8 (44.4)	
Gestational age	<26 weeks	95 (24.0)	6 (33.3)	0.315 *
	26–27 weeks	42 (10.6)	0	
	>27 weeks	259 (65.4)	12 (66.7)	
Birth weight	<700 g	72 (18.2)	6 (33.3)	0.057 *
	700–1200 g	87 (22.0)	6 (33.3)	
	>1200 g	237 (59.8)	6 (33.3)	
Age when PN was started	≤10 days	291 (73.5)	17 (94.4)	0.128 *
	11–28 days	62 (15.7)	0	
	>28 days	43 (10.9)	1 (5.6)	
Vascular device	Central	149 (37.6)	13 (72.2)	0.032 *
	Peripheral	182 (46.0)	3 (16.7)	
	Peripheral changed to central	58 (14.6)	2 (11.1)	
	Central changed to peripheral	7 (1.8)	0	
Fat emulsion	No	246 (62.1)	7 (38.9)	0.048
	Yes	150 (37.9)	11 (61.1)	
Bacteremia	No	301 (76.0)	10 (55.6)	0.089 *
	Yes	95 (24.0)	8 (44.4)	
Multidrug resistance	No	396 (100)	13 (72.2)	<0.001 *
	Yes	0	5 (27.8)	

* Fisher’s Exact test; other analyses were performed using the Chi-square test, and statistically significant results are indicated by bold font. F: Frequency.

**Table 4:** Correlates of multidrug resistance among the 414 admitted neonates.

Variable	Categories	Multidrug Resistance		
		No (n = 409) F (Column%)	Yes (n = 5) F (Column%)	*p* Value
Sex	Female	220 (53.8)	3 (60.0)	1.000 *
	Male	189 (46.2)	2 (40.0)	
Gestational age	<26 weeks	98 (24.0)	3 (60.0)	0.177 *
	26–27 weeks	42 (10.3)	0	
	>27 weeks	269 (65.8)	2 (40.0)	
Birth weight	<700 g	75 (18.3)	3 (60.0)	0.008 *
	700–1200 g	91 (22.2)	2 (40.0)	
	>1200 g	243 (59.4)	0	
Age when PN was started	≤10 days	304 (74.3)	4 (80.0)	0.544 *
	11–28 days	62 (15.2)	0	
	>28 days	43 (10.5)	1 (20.0)	
Vascular device	Central	158 (38.6)	4 (80.0)	0.351 *
	Peripheral	184 (45.0)	1 (20.0)	
	Peripheral changed to central	60 (14.7)	0	
	Central changed to peripheral	7 (1.7)	0	
Fat emulsion	No	158 (38.6)	3 (60.0)	0.381 *
	Yes	251 (61.4)	2 (40.0)	
Bloodstream culture	No	310 (75.8)	1 (20.0)	0.015 *
	Yes	99 (24.2)	4 (80.0)	
Mortality	No	396 (96.8)	0	<0.001 *
	Yes	13 (3.2)	5 (100)	
Cause of death	Other reasons	10 (76.9)	4 (80.0)	1.000 *
	Bacteremia/sepsis/line infection/CLABSI	3 (23.1)	1 (20.0)	

* Fisher’s Exact test; other analyses were performed using the Chi-square test. CLABSI, central line-associated bloodstream infection; F: Frequency.

**Table 5:** Factors associated with positive bloodstream culture among the admitted neonates *.

Variable	Categories	Adjusted OR (95% CI)	*p* Value
Sex	Female	Ref.	0.518
	Male	1.403 (0.503–3.918)	
Gestational age			<0.001
	<26 weeks	70.759 (19.179–261.058)	<0.001
	26–27 weeks	7.098 (1.650–30.537)	0.008
	>27 weeks	Ref.	
Birth weight			<0.001
	<700 g	16.553 (3.443–79.578)	<0.001
	700–1200 g	0.744 (0.196–2.815)	0.663
	>1200 g	Ref.	
Age when PN was started			0.757
	≤10 days	2.024 (0.313–13.088)	0.459
	11–28 days	1.636 (0.194–13.790)	0.651
	>28 days	Ref.	
Vascular device			0.148
	Central	Ref.	
	Peripheral	0.276 (0.073–1.038)	0.057
	Peripheral changed to central	0.162 (0.024–1.099)	0.062
	Central changed to peripheral	0.000	0.999
Fat emulsion	No	Ref.	
	Yes	4.809 (1.728–13.383)	0.003
Multidrug resistance	No	Ref.	
	Yes	56.841 (0.573–5643.274)	0.085
Mortality	No	Ref.	
	Yes	1.346 (0.130–13.897)	0.803
Constant		0.009	<0.001

* Multiple logistic regression.

## Data Availability

The data presented in this study are available on request from the corresponding author. Data were obtained from three hospitals and are available from the authors with the permission of the three hospitals.
